# *Notes from the Field:* Hospital Water Contamination Associated with a Pseudo-Outbreak of *Mycobacterium porcinum* — Wisconsin, 2016–2018

**DOI:** 10.15585/mmwr.mm6849a4

**Published:** 2019-12-13

**Authors:** Heather Kloth, Lina I. Elbadawi, Allen Bateman, Laura Louison, Nijika Shrivastwa

**Affiliations:** **^1^**Bureau of Communicable Diseases, Wisconsin Division of Public Health; ^2^Center for Preparedness and Response, CDC; ^3^Wisconsin State Laboratory of Hygiene.

During January–December 2017, a hospital laboratory in Wisconsin identified a cluster of seven isolates that tested positive for a rapidly growing nontuberculous mycobacterium, *Mycobacterium porcinum*, which is associated with infections of the respiratory tract, bloodstream (caused by pathogen-contaminated intravenous catheters and equipment), surgical sites, and soft tissue (*1*–*3*). All clinical isolates were obtained from respiratory cultures (sputum, bronchoalveolar lavages, or bronchial aspirates) from patients in the hospital’s intensive care units. No associated clinical infections were reported. Because *M. porcinum* is rarely encountered, a concern that these isolates represented laboratory contamination was raised, and the hospital infection prevention team began an internal investigation. During this time, the hospital’s infection prevention team and the Wisconsin State Laboratory of Hygiene (WSLH) investigated possible infection control breaches and laboratory workflow processes. Following the identification of four additional isolates in January 2018, all patient specimens submitted for acid-fast bacteria culture were routed directly to WSLH for testing beginning February 12. WSLH identified three additional positive *M. porcinum* isolates from patients, suggesting that the organism was not a hospital laboratory contaminant. On March 16, the hospital notified the Wisconsin Division of Public Health of the cluster of *M. porcinum*–positive respiratory isolates. By April 12, a total of 20 isolates had been obtained from 16 patients. A retrospective chart review demonstrated that none of the isolates were associated with a clinical infection; other infections accounted for all patients’ illnesses.

Because nontuberculous mycobacteria are found in water, and *M. porcinum* in particular has been recovered from tap water (*1*), the investigation included testing water samples from the ice machines, water dispensers, and handwashing sinks in the intensive care units collected during the week of April 23. *M. porcinum* was subsequently identified during April 30–May 3 in samples obtained from two ice machines and one water dispenser. Inspection of these machines demonstrated visible debris on internal machine parts and dispenser spouts. Since the installation of new machines and parts in June 2018 and revision of the hospital’s cleaning protocols, no further *M. porcinum* patient isolates have been identified. In accordance with a recommendation from the Wisconsin Division of Public Health, staff members at this hospital no longer use tap water when collecting respiratory cultures.

*M. porcinum* is a rapidly growing nontuberculous mycobacterium within the *Mycobacterium fortuitum* complex. Nontuberculous mycobacteria naturally occur in the environment and can be found in soil and water, including potable water systems that supply many U.S. health care facilities (*4*). Nontuberculous mycobacteria have also been associated with outbreaks in health care settings (*1*–*4*). Tap water was used during respiratory specimen collection at the Wisconsin facility and might have contaminated patient specimens. Tap water is not sterile, can lead to false-positive culture results (*4*), and should be avoided when collecting biologic specimens intended for culture. Hospital water management programs should engage clinical partners to ensure safe water use as part of patient care and address maintenance of ice machines and water dispensers within their facilities.
